# Lupus susceptibility region containing *CTLA4* rs17268364 functionally reduces *CTLA4* expression by binding EWSR1 and correlates IFN-α signature

**DOI:** 10.1186/s13075-021-02664-y

**Published:** 2021-11-04

**Authors:** Yuan-yuan Qi, Xin-yu Zhao, Xin-ran Liu, Yan-na Wang, Ya-ling Zhai, Xiao-xue Zhang, Xiao-yang Wang, Li-jie Zhang, Ya-fei Zhao, Yan Cui, Xiang-hui Ning, Xu-jie Zhou

**Affiliations:** 1 Nephrology Hospital, the First Affiliated Hospital of Zhengzhou University, No.1, Jianshe Road, Erqi District, Zhengzhou, Henan 4500052 People’s Republic of China; 2 Institute of Nephrology, Zhengzhou University, No.1, Jianshe Road, Erqi District, Zhengzhou, Henan 4500052 People’s Republic of China; 3 Renal Division, Peking University First Hospital, Peking University Institute of Nephrology, Beijing, China; 4 Department of Urology, the First Affiliated Hospital of Zhengzhou University, Zhengzhou, 4500052 Henan China; 5 Key Laboratory of Renal Disease, Ministry of Health of China, No.8 Xi Shi Ku Street, Xi Cheng District, Beijing, 100034 China; 6 Key Laboratory of Chronic Kidney Disease Prevention and Treatment (Peking University), Ministry of Education, No.8 Xi Shi Ku Street, Xi Cheng District, Beijing, 100034 China

**Keywords:** Systemic lupus erythematosus, Single nucleotide polymorphisms, Immune checkpoint, *CTLA4-ICOS*, rs17268364

## Abstract

**Background:**

Dysregulation of T cells mediated immune responses is a hallmark in the development of systemic lupus erythematosus (SLE). Recent genome wide association study (GWAS) revealed the genetic contribution of variants located in the cytotoxic T lymphocyte-associated protein-4 (*CTLA4*)-inducible T cell co-stimulator (*ICOS*) intergenic region to SLE susceptibility. Our aim is to find a functional variant in this region.

**Methods:**

The genetic association results in the *CTLA4*-*ICOS* region from previous GWAS were adopted to select the potential variant which was further replicated in two independent cohorts (Henan cohort 2053 SLE patients and 1845 healthy controls, Beijing cohort 2303 SLE patients and 19,262 healthy). In order to explore the functional significance in SLE, bioinformatics with validation experiments (including electrophoretic mobility shift assay and luciferase reporter assay) and mRNA expression analysis were also performed.

**Results:**

A variant located in the *CTLA4*-*ICOS* intergenic region, rs17268364, was associated with susceptibility to SLE patients in Chinese populations (risk allele, *p*_meta_ = 7.02×10^−11^, OR 1.19, 95%CI 1.13–1.26). The bioinformatics suggested that rs17268364 might affect the expression of *CTLA4*, not *ICOS*. The rs17268364 risk G allele containing sequence reduced the expression of the reporter gene by binding transcriptional repressor Ewing sarcoma breakpoint region 1 (EWSR1). Following genotype-mRNA expression, the analysis also showed the risk allele of rs17268364 was associated with low *CTLA4* expression in lupus nephritis (LN) patients. Healthy individuals carrying rs17268364 risk G allele was significantly correlated with higher levels of IFN-α signature including increased lymphocyte antigen 6E (*LY6E*) (*p*=0.031), interferon-stimulated gene 15 (*ISG15*) (*p*=0.038), interferon regulatory factor 9 (*IRF9*) (*p*=0.028), and interferon regulatory factor 5 (*IRF5*) (*p*=0.040) mRNA expression.

**Conclusions:**

The present study confirmed the functional role of rs17268364 in the *CTLA4*-*ICOS* intergenic region that increased SLE susceptibility in the Chinese population.

**Supplementary Information:**

The online version contains supplementary material available at 10.1186/s13075-021-02664-y.

## Introduction

Systemic lupus erythematosus (SLE) is a multi-organ involved autoimmune disease characterized as overactivation of T cells followed by B cells proliferation and excessive production of autoantibodies leading to the loss of self-tolerance. Although the pathogenesis of SLE still remains obscure, genetic factors play an essential role. T cell activation-related genetic loci, especially the HLA region, had been confirmed associated with SLE susceptibility by genome wide association studies (GWAS) [1]. Except for the important role of human leukocyte antigen (HLA) in T cell activation, the molecules balancing both T cell activation and inhibition were also crucial participants to generate the tolerance and negative feedback regulation of the immune response. After T cell activation, inducible T cell co-stimulator (ICOS), is constitutively expressed on naïve T lymphocytes contributing to effective T cell-dependent immune responses. Meanwhile, cytotoxic T lymphocyte-associated protein-4 (CTLA4) is also induced and functions as an inhibitory molecule with the activation of T cells. However, the genetic contribution of *CTLA*4 and *ICOS* to SLE susceptibility remains controversial.

*CTLA4* and *ICOS* are located at 2q33 and genetically adjoining. The single nucleotide polymorphisms (SNP)s, rs3087243, rs231725, rs11571302, and rs11571297, located in the *CTLA4*-*ICOS* intergenic region were identified in predisposition to autoimmune diseases including rheumatoid arthritis [2], vitiligo [3], hypothyroidism [4, 5], type 1 diabetes [6–10], and Graves’ disease [11] by GWASs. The SNPs in *CTLA4*-*ICOS* locus were also implicated in relation with SLE susceptibility. Previous genetic association studies mostly focused on the promoter and exon regions of *CTLA4* and *ICOS*. *CTLA4* +49G/A, CT60A/G, −1722T/C, and −318C/T might affect the expression of *CTLA4* [12–14] and was reported associated with the susceptibility to SLE, especially in Asians [15–18]. Several SNPs in *CTLA4* pathway including rs733618 at the 5′ flanking region of *CTLA4* were identified associated with SLE [19]. In contrast, the genetic association studies were limited in *ICOS* gene and the causal variants were believed to be located at the 5’ flanking region of *ICOS* [20]. Despite the considerable contribution of variants in the *CTLA4*-*ICOS* intergenic region to autoimmune diseases, its role has remained unappreciated in SLE.

Recent GWAS identified rs3087243 located in the *CTLA4*-*ICOS* intergenic region was associated with SLE susceptibility after a meta-analysis of Asian and European populations [21]. In light of this inspiring achievement, we aimed to find a functional variant in this region. In the present study, we undertook an evaluation of the *CTLA4*-*ICOS* polymorphisms to SLE susceptibility in the Chinese population. In addition, we also investigated the possible functional mechanisms underlying genetic associations by integrated analysis of bioinformatics using an online database following with experimental validations and transcriptome profiles.

## Methods

### Population

There were two cohorts enrolled in our replication study. The Henan cohort included 2053 patients with SLE and 1845 healthy controls from Middle of China. The Beijing cohort consisted of 2303 SLE patients and 19,262 healthy controls from North of China. The diagnosis of SLE met the American College of Rheumatology revised criteria [22]. Participants in this cohort were geographically and ethnically matched [23], and demographical information of the cohorts was provided in supplementary table S1. Written informed consent was obtained from all the study subjects, and the study was approved by the Ethical Committee of the Medical Ethics Committee of Zhengzhou University First Hospital (2019-KY-134) and the Medical Ethics Committee of Peking University First Hospital (Institutional Review Board approval no. 2020Y158).

### SNP selection

*CTLA*-4 was located in Chromosome 2: 204,732,509–204,738,683, and *ICOS* was located in Chromosome 2: 204,801,471–204,826,300 (Ensembl, GRCh37). The upstream and downstream 10kb of the *CTLA-*4-*ICOS* region spanning 113,791 bp were focused on the present study. A total of 136 SNPs was covered by ImmunoChip [23]. Among 24 SNPs were associated with SLE susceptibility (*p* < 0.05) (detailed genetic association results were extracted from previous publications and were provided in supplementary table S2). The top signals were rs17268364 and rs13029135 with *p* value 1.41×10^−2^ and were in high linkage disequilibrium with *r*^2^ 0.99, D’ 1 according to ASN (East Asian). Since both rs17268364 and rs13029135 are located in intergenic, functional annotations were performed with RegulomeDB which is a database that annotates SNPs with known and predicted regulatory elements in the intergenic regions of the *H. sapiens* genome [24]. Higher RegulomeDB probability score for rs17268364 (rs17268364 = 0.55411; rs13029135 = 0.005) indicated rs17268364 is more likely to be a regulatory variant. Therefore, rs17268364 (risk allele G frequency cases/controls 79.6/74.9, OR 1.41×10^−2^, OR 1.30, 95%CI 1.06–1.61) was selected as the tag SNP. Further bioinformatics were conducted with HaploReg v4.1 for detailed regulatory annotation and GTEx database for expression quantitative trait loci (eQTL) effect associated with *CTLA*-4 expression [25].

### Genotyping

TaqMan real-time PCR was performed and allele-specific probes labeled with fluorochrome VIC or FAM were used to differentiate wild-type and variant alleles. Genotyping was performed using a 7500 Sequence Detection System (Applied Biosystems, Foster City, CA). Additionally, 10% of the DNA specimens were randomly chosen for sequencing. The concordance rate of 100% was seen across the assays.

### Luciferase reporter assay

Luciferase reporter assay was performed as described previously [26]. Sequences of 101 bp flanking rs17268364 were synthesized and subcloned into pGL3-promoter vector (Promega, Madison, WI, USA) using Lipofectamine 2000 (Invitrogen, Carlsbad, CA) according to the instruction of the manufacturer (sequences are shown in Supplementary Table S3). Luciferase reporter assays were determined using the Promega Luciferase Assay System (Promega, Mannheim, Germany).

### Protein mass spectrometry

Nuclear proteins were extracted from HEK 293T nucleus under the instruction of NE-PER Nuclear and Cytoplasmic Extraction Reagents (Thermo Fisher Scientific). Then double-strand DNA probes were synthesized and incubated with the extracted nuclear proteins (Supplementary Table S4). The DNA-protein complexes were added with 10 mM DTT and then 55 mM IAM for reductive alkylation. Followed by incubation with1ug Trypsin for protein digestion overnight at 37 °C. After desalted with C18 column and then dissolved with 15 μL Loading Buffer (0.1% formic acid, 3% acetonitrile), the peptide mixture was collected injected into liquid chromatography-tandem mass spectrometry (LC-MS/MS) (AB SCIEX tripleTOF 5600-plus, Redwood City, CA) platform.

### Electrophoretic mobility shift assay

The sequences of the synthetic double-stranded biotinylated oligonucleotides were provided in Supplementary Table S4. The probes were incubated with the nuclear proteins from HEK 293T cells at room temperature for 30 min. For supershift assays, 10 μl of anti-EWS (catalog no. 11910S; Cell Signaling Technology) antibody was incubated with nuclear proteins from HEK 293T cells for 1 h before adding the relevant labeled probe. The entire reaction mixture was run on a non-denaturing 0.5×TBE 6% polyacrylamide gel for 1h at 60 V at 4°C, transferred onto Biodyne® B nylon membranes (Pall Corporation), and crosslinked at 120 × 100 μJ/cm^2^. Signals were visualized with reagents included in the kit and ChemiDoc XRS (Bio-Rad Laboratories, USA).

### The mRNA expression of CTLA4 and IFN-α signatures

Total RNA was extracted from whole blood and isolated by the TRIzol Reagent (Life Technologies). Whole genome RNA sequencing (RNA-seq) was performed with PE150 (Illumina, San Diego, CA, USA). A total of 99 individuals were enrolled in the RNA-seq project including 57 lupus nephritis (LN) patients, 18 SLE patients (without renal impairment), and 24 healthy controls. Considering the expression of *CTLA4* might be influenced by severe infection, we excluded two individuals with PCT > 0.5 ng/ml (one was 2.23 ng/ml and the other was 3.46 ng/ml).

### Statistical analysis

A goodness-of-fit *χ*^2^ test was used to evaluate the deviation of rs17268364 for the Hardy-Weinberg equilibrium. Allelic and genotypic associations were assessed by a chi-square test to give the odds ratio with a 95% confidence interval. The meta-analysis of the three cohorts (one discovery cohort and two replication cohorts) was conducted by *Mantel-Haenszel* approach. Heterogeneity was performed with Review Manager 5.4. In rs17268364 genotypes and expression analysis, Spearman’s coefficient was calculated in allele-dependent gene expression regulations. Statistical analyses were carried out with the SPSS 13.0 software. Statistical significance was set at *P* < 0.05.

## Result

### The CTLA4-ICOS intergenic polymorphism rs17268364 associated with SLE susceptibility

To investigate the association between rs17268364 in the *CTLA4-ICOS* intergenic region and SLE susceptibility, we validated the genetic association results in the Henan cohort (2053 cases and 1845 controls from Central of China) and Beijing cohort (2303 SLE patients and 19,262 healthy controls from North of China). Deviation from Hardy-Weinberg equilibrium was not observed in SLE patients or controls. The frequency of minor allele A was significantly lower in SLE patients than controls (risk allele cases vs. controls, 78.8% vs. 75.5% in the Henan cohort and 77.8% vs. 75.2% in th eBejing cohort), with *p* value 4.40×10^−4^ (OR 1.21, 95%CI 1.09–1.35) in the Henan cohort and 1.62 × 10^−4^ in the Beijing cohort (OR 1.15, 95%CI 1.07–1.24) (Table 1).

**Table 1:** Association of the rs17268364 with SLE susceptibility

Cohorts (cases/controls)	Population	Risk allele (cases/controls)	*P* value	OR (95%CI)
Beijing ImmunoChip cohort (490/493)	North of China	79.6/74.9	1.41×10^−2^	1.30(1.06–1.61)
Henan replication cohort (2053/1845)	Central of China	78.8/75.5	4.40×10^−4^	1.21(1.09–1.35)
Beijing replication cohort (2303/19262)	North of China	77.8/75.2	1.62×10^−4^	1.15(1.07–1.24)
Meta-analysis^a^ (4846/21600)			7.02×10^−11^	1.19(1.13–1.26)

^a^Heterogeneity *I*^2^ = 0%

In the Chinese population from the previous GWAS cohort [23], the frequency of risk allele G was 79.6 in cases and 74.9 in controls with *p* value 1.41×10^−2^, OR of 1.30, and 95%CI 1.06 to 1.61. After combined analysis, the association result was pronounced with *p* value 7.02×10^− 11^, OR of 1.19, and 95%CI 1.13 to 1.26 (Table 1).

### Regulatory effects of rs17268364 annotated by bioinformatic analysis

To investigate the functional basis of rs17268364 association with SLE etiology, we first checked the potential functional effect of rs17268364 with Encyclopedia of DNA Elements (ENCODE) data available. High-resolution genome wide ChIP-seq profiles for histone modifications showed that rs17268364 was mapped in the promoter region in PMA-I stimulated primary T helper 17 cells and enhancer regions in 6 tissues including primary T cells effector/memory enriched from peripheral blood (HaploReg v4.1 [27], supplementary table S5). Considering the fact that rs17268364 mapped to the H3K4me1, H3K4me3, H3K27ac, and H3K9ac in multiple cell lines, we speculated that rs17268364 might affect the transcription of target gene(s).

### rs17268364 risk G allele reduced reporter gene activity

**Fig. 1:**
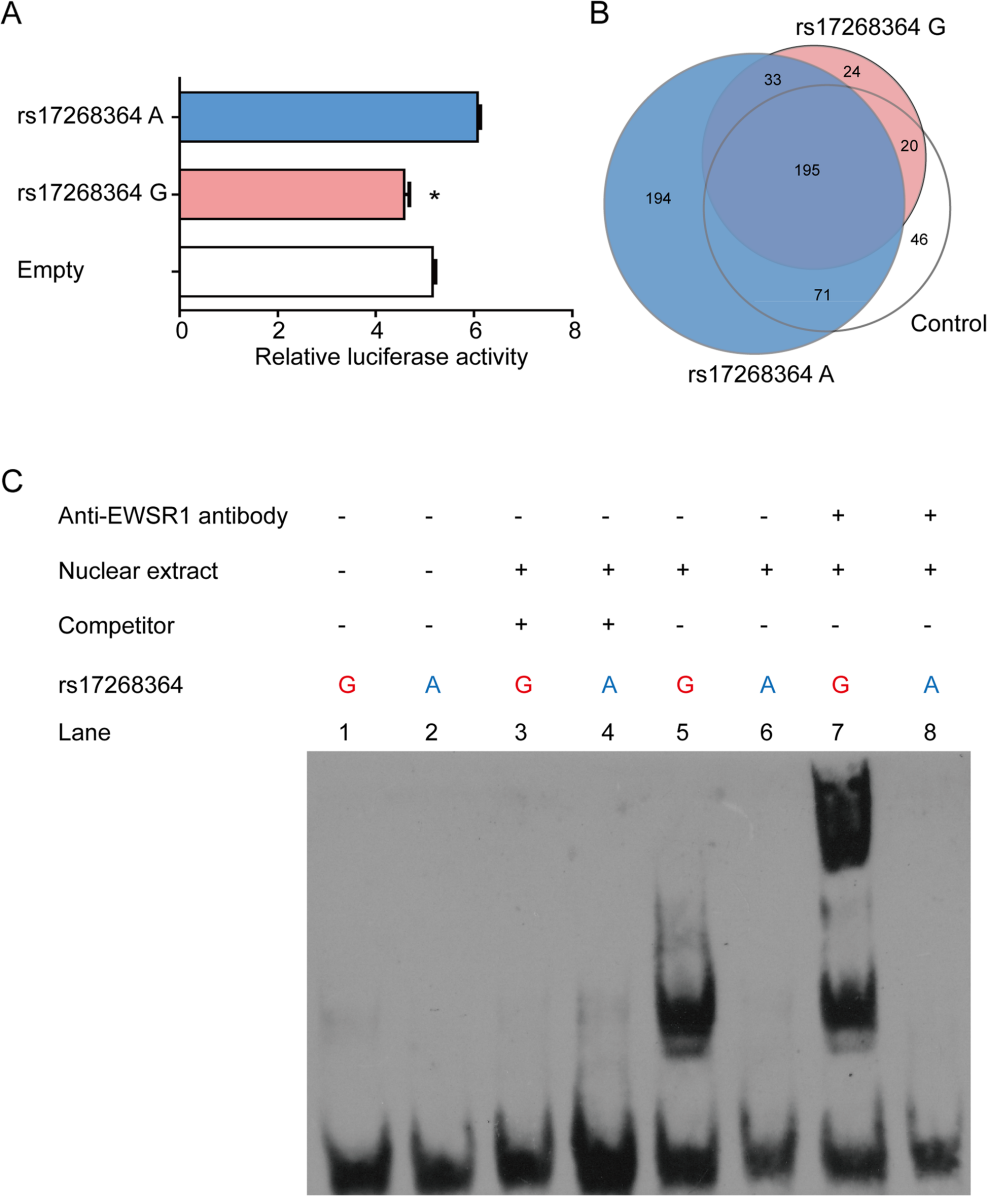
rs17268364 risk G allele binds with EWSR1 and reduced gene expression. **A** In vitro luciferase assays in HEK293T cells showing reduced activity in risk allele constructs containing rs17268364 G allele (*p* = 0.002). **B** Protein mass spectrometry assay and Venn diagram with revealing the proteins pulled down by differentially binding with rs17268364 A or G allele from the lysates of HEK293 T cells. **C** An allele-specific band was observed in probes containing rs17268364 risk G allele. A supershift bind was detected with the rs17268364 risk G allele in addition of an anti-EWSR1 antibody. “+” and “−” mean added and unadded, respectively. Two independent experiments were performed with similar results

To test our hypothesis, the two sequences surrounding rs17268364 containing the protective A allele and risk G allele respectively were synthesized and subcloned into pGL3-promoter vector to test the potential regulatory effect of rs17268364. The results of luciferase activity were presented in Fig. 1A. Comparing with the protective A allele, the rs17268364 risk G allele significantly down-regulated luciferase activity (*p* = 0.002). So, it would be expected that the rs17268364 risk G allele would correlate with a decreased expression of the target gene(s).

### Transcription suppressor EWSR1 binds with rs17268364 risk G allele

In order to identify a potential transcription suppressor that binds rs17268364 risk G allele containing sequence, we performed DNA pulldown coupled with protein mass spectrometry (Fig. 1B). Twenty-four unique proteins were identified binding with rs17268364 risk G allele (Fig. 1B), including Ewing sarcoma breakpoint region 1 (EWSR1) which functions as a transcriptional repressor.

Further allelic differences binding of EWSR1 was validated by electrophoretic mobility shift assay (EMSA) using nuclear protein from 293T cell line. As shown in Fig. 1C, the binding affinities of the DNA protein complex were significantly different depending on the rs17268364 allele. The DNA protein complex binding intensity of the rs17268364 risk G allele was significantly higher than the rs17268364 protective A allele. Furthermore, a supershift bind was detected with the rs17268364 risk G allele in addition to an anti-EWSR1 antibody which confirmed the constitution of the DNA protein complex.

### rs17268364 risk G allele associated with lower CTLA-4 expression by eQTL analysis

**Fig. 2:**
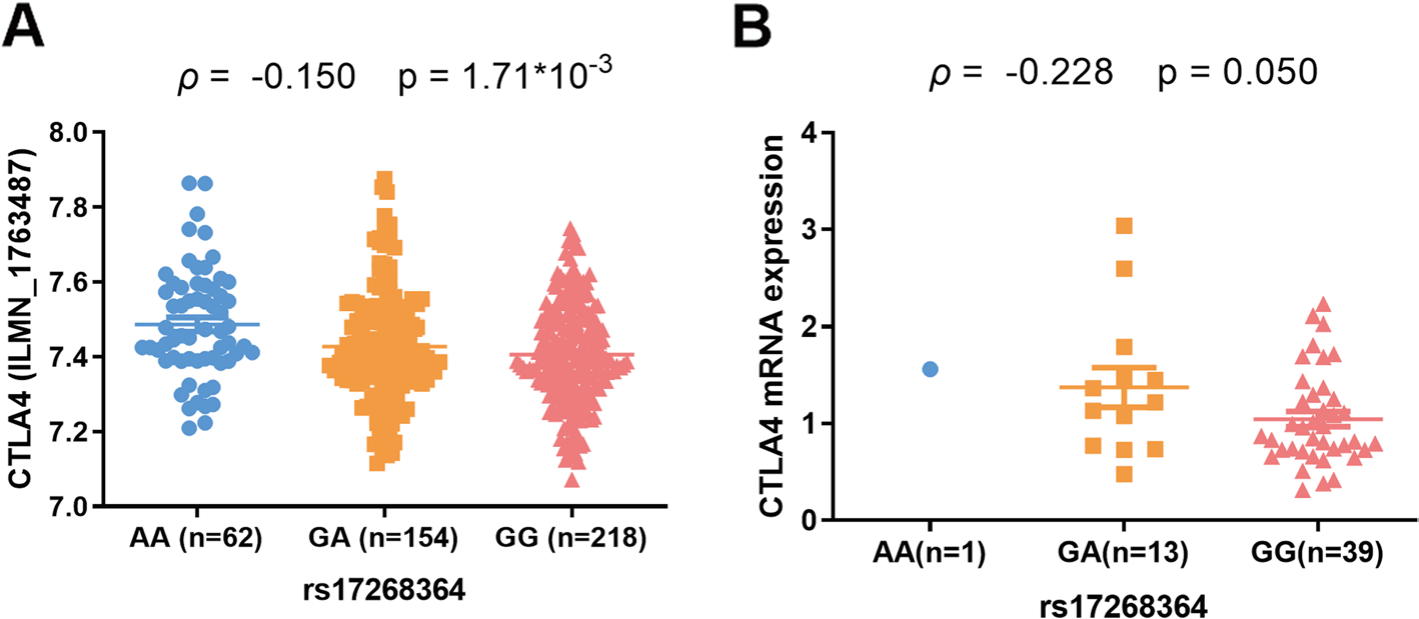
rs17268364 risk G allele associated with lower expression of *CTLA-4.* **A** Integrated analysis of genotype-expression data from HapMap3 individuals including 76 Han Chinese in Beijing, China (CHB), 82 Japanese in Tokyo, Japan (JPT), 90 Yoruba in Ibadan, Nigeria (YRI), 75 Gujarati Indians in Houston, Texas (GIH), 69 Luhya in Webuye, Kenya (LWK), 42 Mexican ancestry in Los Angeles, California (MXL). ILMN_1763487 denotes the probe for *CTLA-4* in the gene expression chip (Illumina). **B** The correlation between mRNA expression of *CTLA-4* and rs17268364 genotypes in lupus nephritis patients

To test this conjecture, we searched the GTEx portal suggesting that rs17268364 was an eQTL locus. A significant correlation between rs17268364 and *CTLA*-4 expression was observed in multiple tissues including testis, esophagus-gastroesophageal junction, artery-aorta, uterus, nerve-tibial, heart-left ventricle, lung, prostate, artery-tibial, and spleen (details in supplementary table S6). The most significant relationship was in testis with elevated expression of *CTLA*-4 and the protective A allele (p = 8.6×10^-12^). Data from GENEVAR database also showed the protective rs17268364 G allele correlated with decreased *CTLA4* expression in Epstein-Barr virus-transformed lymphoblastoid cell lines from 6 HapMap3 populations (*p* = 1.71×10^−3^) (Fig. 2A).

In light of the fact that rs17268364 risk G allele associated with lower *CTLA*-4 expression, we suspected that the SLE patients carrying rs17268364 G allele confer a higher risk of SLE by a low expression of *CTLA*-4 than those carrying rs17268364 A allele.

### rs17268364 risk G allele correlated with lower CTLA-4 expression in SLE patients

The expression of CTLA-4 was strongly influenced by the state of the immune system with a significant upregulation upon immune activation. Thus, to examine our above assumption, we detected the correlation between rs17268364 genotypes and *CTLA*-4 expression in SLE patients and healthy controls respectively (Fig. 2). There was no significant association between rs17268364 genotypes and *CTLA*-4 expression in SLE patients (without renal impairment) or healthy controls (Supplementary Fig. S1). We observed a trend of negative correlation between individuals carrying rs17268364 risk G allele and *CTLA*-4 expression in LN patients (Fig. 2B). No similar results were observed between individuals carrying rs17268364 risk G allele and *ICOS* expression in LN patients (Supplementary Fig. S2).

### rs17268364 risk G allele correlated with higher levels of IFN-α signatures in healthy individuals

**Fig. 3:**
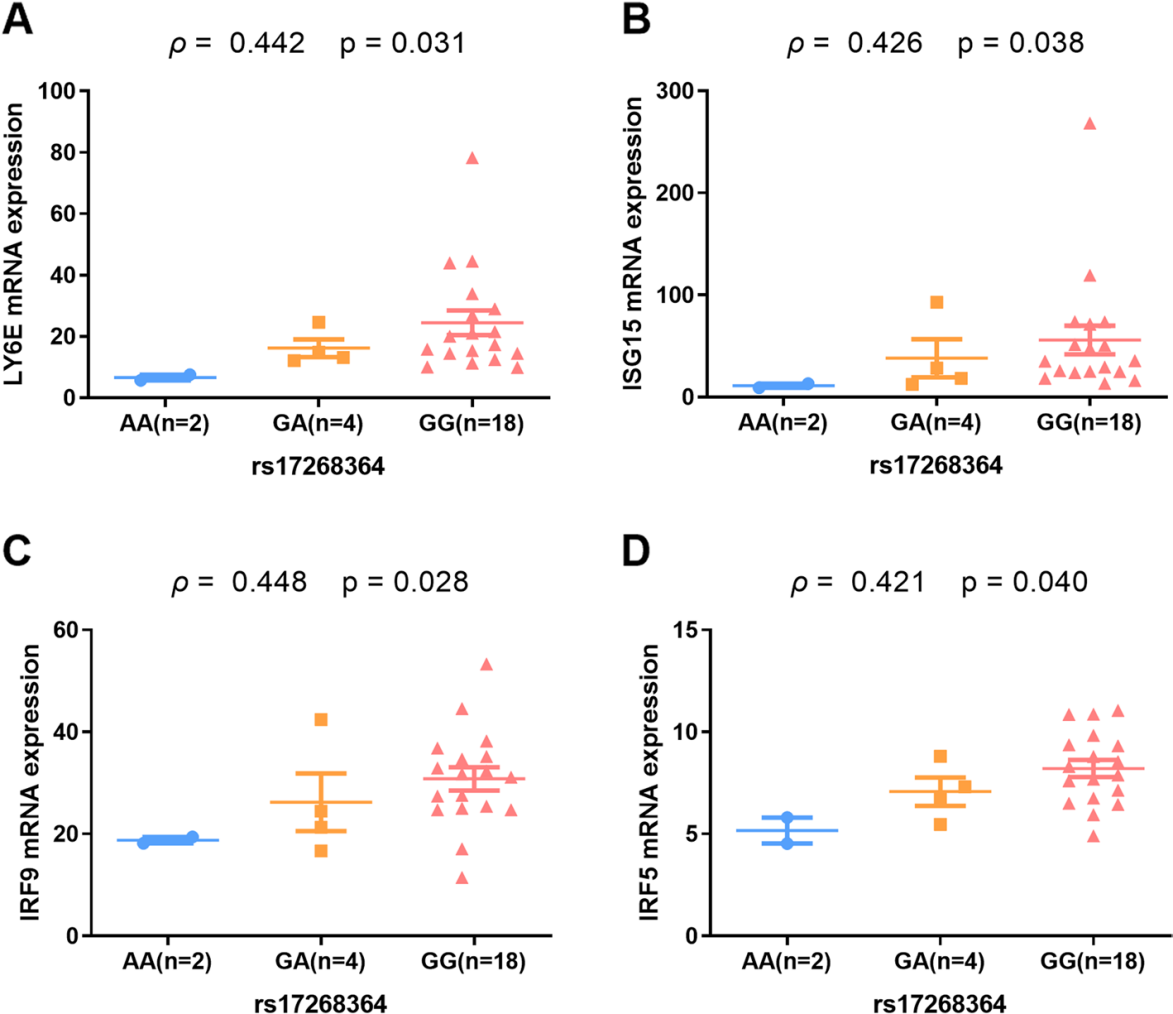
rs17268364 risk G allele associated with higher IFN-α signature. Healthy individuals carrying rs17268364 risk G allele was significantly correlated with increased *LY6E* (*p*=0.031), *ISG15* (*p*=0.038), *IRF9* (*p*=0.028), and *IRF5* (*p*=0.040) mRNA expression. The Spearman’s correlation coefficient (rho) and *p* value for correlation were presented

The critical role of IFN-α signature in the pathogenesis of SLE has been well documented. We further analyzed the levels of IFN-α signature in healthy individuals with rs17268364 A/G genetic background. Healthy individuals carrying rs17268364 risk G allele was significantly correlated with increased lymphocyte antigen 6E (*LY6E*) (*p*=0.031), interferon-stimulated gene 15 (*ISG15*) (*p*=0.038), interferon regulatory factor 9 (*IRF9*) (*p*=0.028), and interferon regulatory factor 5 (*IRF5*) (*p*=0.040) mRNA expression (Fig. 3). This result indicated that healthy individuals with a genetic background of carrying rs17268364 risk G allele might have a higher level of IFN-α signature and were more likely to confer a higher risk of SLE.

## Discussion

In the present study, we first explored the genetic contribution of variants within the *CTLA4*-*ICOS* locus to SLE susceptibility in the Chinese population. According to a previous East Asian ImmunoChip study, the results of genetic association for rs17268364 *CTLA4*-*ICOS* was 1.41×10^−2^ in the Chinese population (490 cases vs. 493 controls from Beijing, North of China) and 7.42×10^−3^ in the Korean population (1710 cases vs. 3167 controls) [23]. The genetic association results from a replication study comprising the Henan cohort (2053 cases and 1845 controls) and Beijing cohort (2303 cases and 19,262 controls) were pronounced with *p* value 4.40×10^−4^ and 1.62×10^−4^ with enlarged sample size. After combined analysis, the result reached the GWAS significance level (1.63×10^−11^) providing robust genetic association between rs17268364 and SLE susceptibility. Although the genetic association between variants at *CTLA4*-*ICOS* locus and SLE susceptibility were hotspots, previous candidate gene studies were mostly focused on the promoter and exon regions of *CTLA4* and *ICOS* leaving the intergenic region yet to be studied. For GWAS and meta GWAS which included rs17268364, sample size and ethnicity might the most two important reasons for missing this finding. However, as an intergenic variant at the *CTLA4*-*ICOS* locus, rs17268364 cannot explain all the association signals of the *CTLA4*-*ICOS* intergenic region (supplementary Fig. S3). More study in this particular region deserves further study. SLE is a complex autoimmune disease with multiple organ damage. We supposed that the association signal from rs17268364 might be diluted by the heterogeneous clinical manifestations of SLE patients. In the future, more clinical manifestations related to genetic signals would be discovered using phenotypic approaches in SLE.

The bioinformatics suggested that rs17268364 *CTLA4*-*ICOS* might affect the expression of *CTLA4*, not *ICOS*. Transcription suppressor EWSR1 binds with rs17268364 risk G allele and luciferase reporter assay demonstrated the risk allele of rs17268364 decreased the expression of reporter gene compared with protective allele. Following genotype-mRNA expression analysis also showed the risk allele of rs17268364 was associated with low *CTLA4* expression in LN patients. Our results implied that risk allele rs17268364 *CTLA4*-*ICOS* might contribute to SLE by reducing the expression of *CTLA4* especially in patients with renal impairment. Defects caused by negative regulators of immune responses were believed to contribute to the overproduction of auto-antibodies and inflammatory factors in patients with SLE. The expression of *CTLA4* was mainly induced after T cell activation by acting as the negative regulator of immune responses. Consistent with previous findings that the deficiency of *CTLA4* was involved in the development of SLE [28], our result showed that the expression of *CTLA4* was not significantly increased in LN patients compared with healthy controls. Moreover, LN patients carrying risk alleles of rs17268364 correlated lower levels of *CTLA4* expression comparing those with protective alleles. The risk allele of rs17268364 might contribute to the deficiency of *CTLA4* expression. These findings might expand our knowledge of *CTLA4* deficiency in the pathogenesis of SLE, particularly in LN.

There were some limitations in two aspects of our study. One is genetic association studies that the population included in our study was predominantly from China. Future investigations with multi-ethnic populations are crucial to generalize the association between rs17268364 and SLE to other populations. Moreover, LN patients constituted the vast majority of cases both in the discovery cohorts and replication cohorts. More cohorts which included both LN and SLE patients without LN were essential to specify whether the genetic association between rs17268364 and SLE was due to LN patients. The other aspect was our expression analysis. We identified that healthy individuals carrying rs17268364 risk G allele correlated with higher levels of IFN-α signatures. However, there were only two individuals who were AA genotype which might lead to a false positive result potentially due to the relatively small sample size. And studies with a larger sample size are necessary to confirm our findings in the future. Interestingly, the marginal correlation between rs17268364 genotypes and *CTLA-4* mRNA expression was observed but not in healthy controls or SLE patients without LN. Enlarged sample size, especially in SLE patients without LN, was crucial to clarify whether the correlation between rs17268364 genotypes and *CTLA-4* mRNA expression was LN specific. What is more, the mRNA expression can be greatly influenced by medication especially steroid and immunosuppressive agents which are commonly used in SLE treatment. SLE patients included in our study were mostly under treatment with steroid or/and immunosuppressive agents leading to a negative correlation in eQTL analysis. The ideal and essential approach is to perform the eQTL analysis in untreated SLE patients which is an important direction for future efforts.

Lupus nephritis remains the main cause of morbidity and mortality in SLE, particularly in Asians [29]. Genetic association analysis and genotypic mRNA expression of *CTLA4* analysis showed a special relation between LN patients and *CTLA4* in our present study. These results corroborate the previous work that CTLA-4-Ig or Abatacept or had shown promising results in alleviating murine lupus nephritis and human lupus nephritis patients after modification of the primary outcome in phase II/III randomized controlled trial of abatacept [30]. In immune checkpoint blockade therapy, target against CTLA-4 induces immune-related adverse events including lupus-like glomerulonephritis [31, 32].

## Conclusions

Here we conducted a combined analysis of genetic association study, bioinformatics with validation, and mRNA expression. A new signal, rs17268364 located in the *CTLA4*-*ICOS* intergenic region, was identified as associated with SLE in the Chinese population. Following investigations further revealed that the risk allele of rs17268364 *CTLA4*-*ICOS* might contribute to SLE by reducing the expression of *CTLA4* especially in patients with renal impairment. The present study provided new insight into variants in the *CTLA4*-*ICOS* intergenic region. Further trans-ethnic dense mapping of the *CTLA4*-*ICOS* intergenic region will be needed.

## Supplementary Information

**Additional file 1: Supplementary table 1.** Demographical information of the cohorts. **Supplementary table 2.** Association results of SNPs in CTLA4-ICOS region and SLE susceptibility (1). **Supplementary Table 3.** Synthesized sequences for subcloning into pGL3-promoter. **Supplementary Table 4.** The sequences of the synthetic double-stranded oligonucleotides for protein mass spectrometry and EMSA. **Supplementary table 5.** Regulatory chromatin states from DNAse and histone ChIP-Seq (Roadmap Epigenomics Consortium, 2015) (2). **Supplementary table 6.** Single-Tissue eQTLs for rs17268364. **Supplementary Figure 1.** The correlation between mRNA expression of CTLA4 and rs17268364 genotypes. A. Healthy controls B. systemic lupus erythematosus patients without renal impairment. **Supplementary Figure 2.** The correlation between mRNA expression of *ICOS* and rs17268364 genotypes in SLE patients without renal impairment (A), lupus nephritis patients (B), and SLE patients (C). **Supplementary Figure 3.** Linkage disequilibrium (LD) heatmap of the 24 identified SLE-associated SNPs. The Linkage disequilibrium (LD) heatmap of the 24 identified SLE-associated SNPs was generated using genotype data of 103 Chinese Han Beijing individuals from 1000 genome project. The degrees of LD were estimated by CI method using Haploview4.2 (Cambridge, MA, USA) and a standard color scheme (D’/LOD) is used to display the LD pattern.

## Data Availability

The data that support the findings of this study are available from the corresponding author upon reasonable request.

## Abbreviations

SLE: Systemic lupus erythematosus
LN: Lupus nephritis
GWAS: Genome wide association study
SNP: Single nucleotide polymorphisms
eQTL: Expression quantitative trait loci
ICOS: Inducible T cell co-stimulator
CTLA4: Cytotoxic T lymphocyte-associated protein-4
EWSR1: Ewing sarcoma breakpoint region 1
LY6E: Lymphocyte antigen 6E
ISG15: Interferon-stimulated gene 15
IRF9: Interferon regulatory factor 9
IRF5: Interferon regulatory factor 5
HLA: Human leukocyte antigen
ENCODE: Encyclopedia of DNA Elements
EMSA: Electrophoretic mobility shift assay
