# Supplementary material for: Lupus susceptibility region containing CTLA4 rs17268364 functionally reduces CTLA4 expression by binding EWSR1 and correlates IFN-α signature
Source: Arthritis Res Ther. 2021 Nov 4;23:279. doi: 10.1186/s13075-021-02664-y (PMC8567630; doi:10.1186/s13075-021-02664-y)
Supplement: Supplementary file 1 — Additional file 1: Supplementary table 1. Demographical information of the cohorts. Supplementary table 2. Association results of SNPs in CTLA4-ICOS region and SLE susceptibility (1). Supplementary Table 3. Synthesized sequences for subcloning into pGL3-promoter. Supplementary Table 4. The sequences of the synthetic double-stranded oligonucleotides for protein mass spectrometry and EMSA. Supplementary table 5. Regulatory chromatin states from DNAse and histone ChIP-Seq (Roadmap Epigenomics Consortium, 2015) (2). Supplementary table 6. Single-Tissue eQTLs for rs17268364. Supplementary Figure 1. The correlation between mRNA expression of CTLA4 and rs17268364 genotypes. A. Healthy controls B. systemic lupus erythematosus patients without renal impairment. Supplementary Figure 2. The correlation between mRNA expression of ICOS and rs17268364 genotypes in SLE patients without renal impairment (A), lupus nephritis patients (B), and SLE patients (C). Supplementary Figure 3. Linkage disequilibrium (LD) heatmap of the 24 identified SLE-associated SNPs. The Linkage disequilibrium (LD) heatmap of the 24 identified SLE-associated SNPs was generated using genotype data of 103 Chinese Han Beijing individuals from 1000 genome project. The degrees of LD were estimated by CI method using Haploview4.2 (Cambridge, MA, USA) and a standard color scheme (D’/LOD) is used to display the LD pattern. [file 13075_2021_2664_MOESM1_ESM.zip › supplementary files - clean version.docx]

**Lupus Susceptibility Region Containing *CTLA4* rs17268364** **Functionally Reduces *CTLA4* Expression by Binding EWSR1 and Correlates IFN-α Signature**

Yuan-yuan Qi^12#*^, Xin-yu Zhao^#12^, Xin-ran Liu^12#^, Yan-na Wang^3^, Ya-ling Zhai^12^, Xiao-xue Zhang^12^, Xiao-yang Wang^12^, Li-jie Zhang^12^, Ya-fei Zhao^12^, Yan Cui^12^, Xiang-hui Ning^4^, Xu-jie Zhou^3*^

**AUTHORS’ INSTITUTION AND AFFILIATION**

1.Nephrology Hospital, the First Affiliated Hospital of Zhengzhou University, Henan 4500052, China;

2. Institute of Nephrology, Zhengzhou University, Henan 4500052, China;

3. Renal Division, Peking University First Hospital, Peking University Institute of Nephrology, Beijing, People’s Republic of China

4. Department of Urology, the First Affiliated Hospital of Zhengzhou University, Henan 4500052, China.

^#^: These authors contribute equally to this work.

CORRESPONDING AUTHOR

Dr. Yuan-yuan Qi, MD & PhD;

Email: qqyyiillyy@126.com

Nephrology Hospital, the First Affiliated Hospital of Zhengzhou University,

Institute of Nephrology, Zhengzhou University

No.1, Jianshe Road, Erqi District

Zhengzhou 4500052, P.R China

Or

Dr. Xu-jie Zhou, MD & PhD;

Email: zhouxujie@bjmu.edu.cn

Renal Division, Department of Medicine, Peking University First Hospital;
Peking University Institute of Nephrology
Key Laboratory of Renal Disease, Ministry of Health of China;
Key Laboratory of Chronic Kidney Disease Prevention and Treatment (Peking University), Ministry of Education;
No.8 Xi Shi Ku Street, Xi Cheng District
Beijing 100034, P.R. China
Tel. +86-10-83572388,
Fax +86-10-66551055

**KEY WORDS**

Systemic lupus erythematosus, Single nucleotide polymorphisms, immune checkpoint, *CTLA4-ICOS,* rs17268364

Supplementary table 1. Demographical information of the cohorts

| Cohorts |  | Female (%) | Age (mean±SD, year) | Lupus nephritis (%) |
| --- | --- | --- | --- | --- |
| Beijing ImmunoChip cohort | Cases (n=490) | 419 (85.5) | - | 2303 (100) |
|  | Controls (n=493) | 356 (72.2) | - | - |
|  |  |  |  |  |
| Henan replication cohort | Cases (n=2053) | 1871(91.1) | 35.41±13.29 | 1143(55.7) |
|  | Controls (n=1845) | 856(46.4) | 45.78±15.31 | - |
|  |  |  |  |  |
| Beijing replication cohort | Cases (n=2303) | 2057(89.3) | 33.44±12.64 | 2303 (100) |
|  | Controls (n=19262) | 10790(56.1) | 35.84±12.44 | - |

Supplementary table 2. Association results of SNPs in CTLA4-ICOS region and SLE susceptibility ([1](#_ENREF_1)).

| SNP | Chr. | Position (hg19) | Minor Allele | MAF (case/control, %) | *P*-value | OR (95%CI) | GENCODE genes |
| --- | --- | --- | --- | --- | --- | --- | --- |
| rs733618 | 2 | 204730944 | C | 41.8/37.4 | 4.55*10^-2^ | 1.20(1.00-1.44) | 1.6kb 5' of CTLA4 |
| rs3087243 | 2 | 204738919 | A | 14.9/18.9 | 1.89*10^-2^ | 0.75(0.59-0.95) | 235bp 3' of CTLA4 |
| rs11571302 | 2 | 204742934 | T | 17.8/22.0 | 1.82*10^-2^ | 0.77(0.61-0.96) | 4.3kb 3' of CTLA4 |
| rs11571297 | 2 | 204745003 | C | 18.0/22.4 | 1.51*10^-2^ | 0.76(0.61-0.95) | 6.3kb 3' of CTLA4 |
| rs960792 | 2 | 204749250 | C | 19.6/23.7 | 2.59*10^-2^ | 0.78(0.63-0.97) | 11kb 3' of CTLA4 |
| rs78960870 | 2 | 204749917 | C | 8.1/10.7 | 4.89*10^-2^ | 0.74(0.54-1.00) | 11kb 3' of CTLA4 |
| rs7600322 | 2 | 204754353 | C | 19.6/23.7 | 2.59*10^-2^ | 0.78(0.63-0.97) | 16kb 3' of CTLA4 |
| rs6748358 | 2 | 204756905 | A | 19.6/23.7 | 2.59*10^-2^ | 0.78(0.63-0.97) | 18kb 3' of CTLA4 |
| rs17268364 | 2 | 204777818 | A | 20.4/25.1 | 1.41*10^-2^ | 0.77(0.62-0.95) | 24kb 5' of ICOS |
| rs3116521 | 2 | 204780841 | C | 20.4/25.0 | 1.62*10^-2^ | 0.77(0.62-0.95) | 21kb 5' of ICOS |
| rs62182631 | 2 | 204781918 | G | 20.4/25.0 | 1.62*10^-2^ | 0.77(0.62-0.95) | 20kb 5' of ICOS |
| rs7596727 | 2 | 204783582 | C | 20.4/25.0 | 1.52*10^-2^ | 0.77(0.62-0.95) | 18kb 5' of ICOS |
| rs13029135 | 2 | 204784212 | A | 20.4/25.1 | 1.41*10^-2^ | 0.77(0.62-0.95) | 17kb 5' of ICOS |
| rs10932027 | 2 | 204786474 | A | 20.5/25.1 | 1.64*10^-2^ | 0.77(0.62-0.95) | 15kb 5' of ICOS |
| rs62182632 | 2 | 204787134 | T | 20.5/25.1 | 1.64*10^-2^ | 0.77(0.62-0.95) | 14kb 5' of ICOS |
| rs2033171 | 2 | 204788156 | C | 20.5/25.1 | 1.64*10^-2^ | 0.77(0.62-0.95) | 13kb 5' of ICOS |
| rs11571307 | 2 | 204790611 | C | 20.4/25.0 | 1.62*10^-2^ | 0.77(0.62-0.95) | 11kb 5' of ICOS |
| rs1978595 | 2 | 204791529 | T | 20.6/25.1 | 1.91*10^-2^ | 0.78(0.63-0.96) | 9.9kb 5' of ICOS |
| rs1896493 | 2 | 204792409 | A | 20.2/24.2 | 3.18*10^-2^ | 0.79(0.64-0.98) | 9.1kb 5' of ICOS |
| rs10932028 | 2 | 204793063 | A | 20.3/24.5 | 2.61*10^-2^ | 0.79(0.63-0.97) | 8.4kb 5' of ICOS |
| rs11571310 | 2 | 204793298 | T | 20.2/24.4 | 2.41*10^-2^ | 0.78(0.63-0.97) | 8.2kb 5' of ICOS |
| rs11571309 | 2 | 204793339 | T | 20.2/24.3 | 2.75*10^-2^ | 0.79(0.64-0.97) | 8.1kb 5' of ICOS |
| rs11571306 | 2 | 204799102 | C | 51.6/47.1 | 4.25*10^-2^ | 1.20(1.01-1.43) | 2.4kb 5' of ICOS |
| rs11571305 | 2 | 204800126 | A | 51.5/47.0 | 4.26*10^-2^ | 1.20(1.01-1.43) | 1.3kb 5' of ICOS |

Supplementary Table 3. Synthesized sequences for subcloning into pGL3-promoter.

| SNP | Sequences |
| --- | --- |
| rs17268364 A | TCCAAGTGCTCAGAAGCCATGTGTGTGCCAGGGGCAGATGCAGTGGTGAG**A**ACCTCATCCAGGGACCCAGTGACTCCTTGACAGCATTTTTCCGGGTTGGC  GCCAACCCGGAAAAATGCTGTCAAGGAGTCACTGGGTCCCTGGATGAGGTTCTCACCACTGCATCTGCCCCTGGCACACACATGGCTTCTGAGCACTTGGA |
|  |  |
| rs17268364 G | TCCAAGTGCTCAGAAGCCATGTGTGTGCCAGGGGCAGATGCAGTGGTGAG**G**ACCTCATCCAGGGACCCAGTGACTCCTTGACAGCATTTTTCCGGGTTGGC  GCCAACCCGGAAAAATGCTGTCAAGGAGTCACTGGGTCCCTGGATGAGGTCCTCACCACTGCATCTGCCCCTGGCACACACATGGCTTCTGAGCACTTGGA |
|  |  |

Supplementary Table 4. The sequences of the synthetic double-stranded oligonucleotides for protein mass spectrometry and EMSA

| SNP | Sequences |
| --- | --- |
| rs17268364 A | CAGTGGTGAG**A**ACCTCATCCA  TGGATGAGGTTCTCACCACTG |
| rs17268364 G | CAGTGGTGAG**G**ACCTCATCCA  TGGATGAGGTCCTCACCACTG |

Supplementary table 5. Regulatory chromatin states from DNAse and histone ChIP-Seq (Roadmap Epigenomics Consortium, 2015)([2](#_ENREF_2))

| **Epigenome ID (EID)** | **Group** | **Mnemonic** | **Description** | **Chromatin states** | **Chromatin states** | **H3K4me1** | **H3K4me3** | **H3K27ac** | **H3K9ac** | **DNase** |
| --- | --- | --- | --- | --- | --- | --- | --- | --- | --- | --- |
|  |  |  |  | **(Core 15-state model)** | **(25-state model** |  |  |  |  |  |
|  |  |  |  |  | **using 12 imputed marks)** |  |  |  |  |  |
| E017 | IMR90 | LNG.IMR90 | IMR90 fetal lung fibroblasts Cell Line |  |  |  |  |  |  |  |
| E002 | ESC | ESC.WA7 | ES-WA7 Cells |  |  |  |  |  |  |  |
| E008 | ESC | ESC.H9 | H9 Cells |  |  |  |  |  |  |  |
| E001 | ESC | ESC.I3 | ES-I3 Cells |  |  |  |  |  |  |  |
| E015 | ESC | ESC.HUES6 | HUES6 Cells |  |  |  |  |  |  |  |
| E014 | ESC | ESC.HUES48 | HUES48 Cells |  |  |  |  |  |  |  |
| E016 | ESC | ESC.HUES64 | HUES64 Cells |  |  |  |  |  |  |  |
| E003 | ESC | ESC.H1 | H1 Cells |  |  |  |  |  |  |  |
| E024 | ESC | ESC.4STAR | ES-UCSF4 Cells |  |  |  |  |  |  |  |
| E020 | iPSC | IPSC.20B | iPS-20b Cells |  |  |  |  |  |  |  |
| E019 | iPSC | IPSC.18 | iPS-18 Cells |  |  |  |  |  |  |  |
| E018 | iPSC | IPSC.15b | iPS-15b Cells |  |  |  |  |  |  |  |
| E021 | iPSC | IPSC.DF.6.9 | iPS DF 6.9 Cells |  |  |  |  |  |  |  |
| E022 | iPSC | IPSC.DF.19.11 | iPS DF 19.11 Cells |  |  |  |  |  |  |  |
| E007 | ES-deriv | ESDR.H1.NEUR.PROG | H1 Derived Neuronal Progenitor Cultured Cells |  |  |  |  |  |  |  |
| E009 | ES-deriv | ESDR.H9.NEUR.PROG | H9 Derived Neuronal Progenitor Cultured Cells |  |  |  |  |  |  |  |
| E010 | ES-deriv | ESDR.H9.NEUR | H9 Derived Neuron Cultured Cells |  |  |  |  |  |  |  |
| E013 | ES-deriv | ESDR.CD56.MESO | hESC Derived CD56+ Mesoderm Cultured Cells |  |  |  |  |  |  |  |
| E012 | ES-deriv | ESDR.CD56.ECTO | hESC Derived CD56+ Ectoderm Cultured Cells |  |  | H3K4me1_Enh |  |  |  |  |
| E011 | ES-deriv | ESDR.CD184.ENDO | hESC Derived CD184+ Endoderm Cultured Cells |  |  |  |  | H3K27ac_Enh |  |  |
| E004 | ES-deriv | ESDR.H1.BMP4.MESO | H1 BMP4 Derived Mesendoderm Cultured Cells |  |  |  |  |  |  |  |
| E005 | ES-deriv | ESDR.H1.BMP4.TROP | H1 BMP4 Derived Trophoblast Cultured Cells |  |  |  |  |  |  |  |
| E006 | ES-deriv | ESDR.H1.MSC | H1 Derived Mesenchymal Stem Cells |  |  |  |  |  |  |  |
| E062 | Blood & T-cell | BLD.PER.MONUC.PC | Primary mononuclear cells from peripheral blood |  |  |  |  |  |  |  |
| E034 | Blood & T-cell | BLD.CD3.PPC | Primary T cells from peripheral blood |  |  |  |  |  |  |  |
| E045 | Blood & T-cell | BLD.CD4.CD25I.CD127.TMEMPC | Primary T cells effector/memory enriched from peripheral blood |  |  | H3K4me1_Enh |  |  |  |  |
| E033 | Blood & T-cell | BLD.CD3.CPC | Primary T cells from cord blood |  |  |  |  |  |  |  |
| E044 | Blood & T-cell | BLD.CD4.CD25.CD127M.TREGPC | Primary T regulatory cells from peripheral blood |  |  |  |  |  |  |  |
| E043 | Blood & T-cell | BLD.CD4.CD25M.TPC | Primary T helper cells from peripheral blood |  |  |  |  |  |  |  |
| E039 | Blood & T-cell | BLD.CD4.CD25M.CD45RA.NPC | Primary T helper naive cells from peripheral blood |  |  |  |  |  |  |  |
| E041 | Blood & T-cell | BLD.CD4.CD25M.IL17M.PL.TPC | Primary T helper cells PMA-I stimulated |  |  |  |  |  |  |  |
| E042 | Blood & T-cell | BLD.CD4.CD25M.IL17P.PL.TPC | Primary T helper 17 cells PMA-I stimulated |  |  |  | H3K4me3_Pro |  |  |  |
| E040 | Blood & T-cell | BLD.CD4.CD25M.CD45RO.MPC | Primary T helper memory cells from peripheral blood 1 |  |  |  |  |  |  |  |
| E037 | Blood & T-cell | BLD.CD4.MPC | Primary T helper memory cells from peripheral blood 2 |  |  |  |  |  |  |  |
| E048 | Blood & T-cell | BLD.CD8.MPC | Primary T CD8+ memory cells from peripheral blood |  |  |  |  |  |  |  |
| E038 | Blood & T-cell | BLD.CD4.NPC | Primary T helper naive cells from peripheral blood |  |  |  |  |  |  |  |
| E047 | Blood & T-cell | BLD.CD8.NPC | Primary T CD8+ naive cells from peripheral blood |  |  |  |  |  |  |  |
| E029 | HSC & B-cell | BLD.CD14.PC | Primary monocytes from peripheral blood |  |  |  |  |  |  |  |
| E031 | HSC & B-cell | BLD.CD19.CPC | Primary B cells from cord blood |  |  |  |  |  |  |  |
| E035 | HSC & B-cell | BLD.CD34.PC | Primary hematopoietic stem cells |  |  |  |  |  |  |  |
| E051 | HSC & B-cell | BLD.MOB.CD34.PC.M | Primary hematopoietic stem cells G-CSF-mobilized Male |  |  |  |  |  |  |  |
| E050 | HSC & B-cell | BLD.MOB.CD34.PC.F | Primary hematopoietic stem cells G-CSF-mobilized Female |  |  |  |  |  |  |  |
| E036 | HSC & B-cell | BLD.CD34.CC | Primary hematopoietic stem cells short term culture |  |  |  |  |  |  |  |
| E032 | HSC & B-cell | BLD.CD19.PPC | Primary B cells from peripheral blood |  |  |  |  |  |  |  |
| E046 | HSC & B-cell | BLD.CD56.PC | Primary Natural Killer cells from peripheral blood |  |  |  |  |  |  |  |
| E030 | HSC & B-cell | BLD.CD15.PC | Primary neutrophils from peripheral blood |  |  |  |  |  |  |  |
| E026 | Mesench | STRM.MRW.MSC | Bone Marrow Derived Cultured Mesenchymal Stem Cells |  |  |  |  |  |  |  |
| E049 | Mesench | STRM.CHON.MRW.DR.MSC | Mesenchymal Stem Cell Derived Chondrocyte Cultured Cells |  |  |  |  |  |  |  |
| E025 | Mesench | FAT.ADIP.DR.MSC | Adipose Derived Mesenchymal Stem Cell Cultured Cells |  |  |  |  |  |  |  |
| E023 | Mesench | FAT.MSC.DR.ADIP | Mesenchymal Stem Cell Derived Adipocyte Cultured Cells |  |  |  |  |  |  |  |
| E052 | Myosat | MUS.SAT | Muscle Satellite Cultured Cells |  |  |  |  |  |  |  |
| E055 | Epithelial | SKIN.PEN.FRSK.FIB.01 | Foreskin Fibroblast Primary Cells skin01 |  |  |  |  |  |  |  |
| E056 | Epithelial | SKIN.PEN.FRSK.FIB.02 | Foreskin Fibroblast Primary Cells skin02 |  |  |  |  |  |  |  |
| E059 | Epithelial | SKIN.PEN.FRSK.MEL.01 | Foreskin Melanocyte Primary Cells skin01 |  |  |  |  |  |  |  |
| E061 | Epithelial | SKIN.PEN.FRSK.MEL.03 | Foreskin Melanocyte Primary Cells skin03 |  |  |  |  |  |  |  |
| E057 | Epithelial | SKIN.PEN.FRSK.KER.02 | Foreskin Keratinocyte Primary Cells skin02 |  |  |  |  |  |  |  |
| E058 | Epithelial | SKIN.PEN.FRSK.KER.03 | Foreskin Keratinocyte Primary Cells skin03 |  |  |  |  |  |  |  |
| E028 | Epithelial | BRST.HMEC.35 | Breast variant Human Mammary Epithelial Cells (vHMEC) |  |  |  |  |  |  |  |
| E027 | Epithelial | BRST.MYO | Breast Myoepithelial Primary Cells |  |  |  |  |  | H3K9ac_Pro |  |
| E054 | Neurosph | BRN.GANGEM.DR.NRSPHR | Ganglion Eminence derived primary cultured neurospheres |  |  |  |  |  |  |  |
| E053 | Neurosph | BRN.CRTX.DR.NRSPHR | Cortex derived primary cultured neurospheres |  |  |  |  |  |  |  |
| E112 | Thymus | THYM | Thymus |  |  |  |  |  |  |  |
| E093 | Thymus | THYM.FET | Fetal Thymus |  |  |  |  |  |  |  |
| E071 | Brain | BRN.HIPP.MID | Brain Hippocampus Middle |  |  |  |  |  |  |  |
| E074 | Brain | BRN.SUB.NIG | Brain Substantia Nigra |  |  |  |  |  |  |  |
| E068 | Brain | BRN.ANT.CAUD | Brain Anterior Caudate |  |  |  |  |  |  |  |
| E069 | Brain | BRN.CING.GYR | Brain Cingulate Gyrus |  |  |  |  |  |  |  |
| E072 | Brain | BRN.INF.TMP | Brain Inferior Temporal Lobe |  |  |  |  |  |  |  |
| E067 | Brain | BRN.ANG.GYR | Brain Angular Gyrus |  |  |  |  |  |  |  |
| E073 | Brain | BRN.DL.PRFRNTL.CRTX | Brain_Dorsolateral_Prefrontal_Cortex |  |  |  |  |  |  |  |
| E070 | Brain | BRN.GRM.MTRX | Brain Germinal Matrix |  |  |  |  |  |  |  |
| E082 | Brain | BRN.FET.F | Fetal Brain Female |  |  |  |  |  |  |  |
| E081 | Brain | BRN.FET.M | Fetal Brain Male |  |  |  |  |  |  |  |
| E063 | Adipose | FAT.ADIP.NUC | Adipose Nuclei |  |  | H3K4me1_Enh |  |  | H3K9ac_Pro |  |
| E100 | Muscle | MUS.PSOAS | Psoas Muscle |  |  |  |  |  |  |  |
| E108 | Muscle | MUS.SKLT.F | Skeletal Muscle Female |  |  |  |  |  |  |  |
| E107 | Muscle | MUS.SKLT.M | Skeletal Muscle Male |  |  |  |  |  |  |  |
| E089 | Muscle | MUS.TRNK.FET | Fetal Muscle Trunk |  |  | H3K4me1_Enh |  |  |  |  |
| E090 | Muscle | MUS.LEG.FET | Fetal Muscle Leg |  |  |  |  |  |  |  |
| E083 | Heart | HRT.FET | Fetal Heart |  |  |  |  |  |  |  |
| E104 | Heart | HRT.ATR.R | Right Atrium |  |  |  |  |  |  |  |
| E095 | Heart | HRT.VENT.L | Left Ventricle |  |  | H3K4me1_Enh |  |  |  |  |
| E105 | Heart | HRT.VNT.R | Right Ventricle |  |  |  |  | H3K27ac_Enh |  |  |
| E065 | Heart | VAS.AOR | Aorta |  |  |  |  |  |  |  |
| E078 | Sm. Muscle | GI.DUO.SM.MUS | Duodenum Smooth Muscle |  |  |  |  |  |  |  |
| E076 | Sm. Muscle | GI.CLN.SM.MUS | Colon Smooth Muscle |  |  |  |  |  |  |  |
| E103 | Sm. Muscle | GI.RECT.SM.MUS | Rectal Smooth Muscle |  |  |  |  |  |  |  |
| E111 | Sm. Muscle | GI.STMC.MUS | Stomach Smooth Muscle |  |  |  |  |  |  |  |
| E092 | Digestive | GI.STMC.FET | Fetal Stomach |  |  | H3K4me1_Enh |  |  |  |  |
| E085 | Digestive | GI.S.INT.FET | Fetal Intestine Small |  |  |  |  |  |  |  |
| E084 | Digestive | GI.L.INT.FET | Fetal Intestine Large |  |  |  |  |  |  |  |
| E109 | Digestive | GI.S.INT | Small Intestine |  |  |  |  |  |  |  |
| E106 | Digestive | GI.CLN.SIG | Sigmoid Colon |  |  |  |  |  |  |  |
| E075 | Digestive | GI.CLN.MUC | Colonic Mucosa |  |  |  |  |  |  |  |
| E101 | Digestive | GI.RECT.MUC.29 | Rectal Mucosa Donor 29 |  |  |  |  |  |  |  |
| E102 | Digestive | GI.RECT.MUC.31 | Rectal Mucosa Donor 31 |  |  |  |  |  |  |  |
| E110 | Digestive | GI.STMC.MUC | Stomach Mucosa |  |  |  |  |  |  |  |
| E077 | Digestive | GI.DUO.MUC | Duodenum Mucosa |  |  |  |  |  |  |  |
| E079 | Digestive | GI.ESO | Esophagus |  |  |  |  |  |  |  |
| E094 | Digestive | GI.STMC.GAST | Gastric |  |  |  |  |  |  |  |
| E099 | Other | PLCNT.AMN | Placenta Amnion |  |  |  |  |  |  |  |
| E086 | Other | KID.FET | Fetal Kidney |  |  |  |  |  |  |  |
| E088 | Other | LNG.FET | Fetal Lung |  |  |  |  |  |  |  |
| E097 | Other | OVRY | Ovary |  |  |  |  |  |  |  |
| E087 | Other | PANC.ISLT | Pancreatic Islets |  |  |  |  |  |  |  |
| E080 | Other | ADRL.GLND.FET | Fetal Adrenal Gland |  |  |  |  |  |  |  |
| E091 | Other | PLCNT.FET | Placenta |  |  |  |  |  |  |  |
| E066 | Other | LIV.ADLT | Liver |  |  |  |  |  |  |  |
| E098 | Other | PANC | Pancreas |  |  |  |  |  |  |  |
| E096 | Other | LNG | Lung |  |  |  |  |  |  |  |
| E113 | Other | SPLN | Spleen |  |  |  |  |  |  |  |
| E114 | ENCODE2012 | LNG.A549.ETOH002.CNCR | A549 EtOH 0.02pct Lung Carcinoma Cell Line |  |  |  |  |  |  |  |
| E115 | ENCODE2012 | BLD.DND41.CNCR | Dnd41 TCell Leukemia Cell Line |  |  |  |  |  |  |  |
| E116 | ENCODE2012 | BLD.GM12878 | GM12878 Lymphoblastoid Cells |  |  |  |  |  |  |  |
| E117 | ENCODE2012 | CRVX.HELAS3.CNCR | HeLa-S3 Cervical Carcinoma Cell Line |  |  |  |  |  |  |  |
| E118 | ENCODE2012 | LIV.HEPG2.CNCR | HepG2 Hepatocellular Carcinoma Cell Line |  |  |  |  |  |  |  |
| E119 | ENCODE2012 | BRST.HMEC | HMEC Mammary Epithelial Primary Cells |  |  |  |  |  |  |  |
| E120 | ENCODE2012 | MUS.HSMM | HSMM Skeletal Muscle Myoblasts Cells |  |  |  |  |  |  |  |
| E121 | ENCODE2012 | MUS.HSMMT | HSMM cell derived Skeletal Muscle Myotubes Cells |  |  |  |  |  |  |  |
| E122 | ENCODE2012 | VAS.HUVEC | HUVEC Umbilical Vein Endothelial Primary Cells |  |  |  |  |  |  |  |
| E123 | ENCODE2012 | BLD.K562.CNCR | K562 Leukemia Cells |  |  |  |  |  |  |  |
| E124 | ENCODE2012 | BLD.CD14.MONO | Monocytes-CD14+ RO01746 Primary Cells |  |  |  |  |  |  |  |
| E125 | ENCODE2012 | BRN.NHA | NH-A Astrocytes Primary Cells |  |  |  |  |  |  |  |
| E126 | ENCODE2012 | SKIN.NHDFAD | NHDF-Ad Adult Dermal Fibroblast Primary Cells |  |  |  |  |  |  |  |
| E127 | ENCODE2012 | SKIN.NHEK | NHEK-Epidermal Keratinocyte Primary Cells |  |  |  |  |  |  |  |
| E128 | ENCODE2012 | LNG.NHLF | NHLF Lung Fibroblast Primary Cells |  |  |  |  |  |  |  |
| E129 | ENCODE2012 | BONE.OSTEO | Osteoblast Primary Cells |  |  |  |  |  |  |  |
| This table was adopted from HaploReg v4.1. Black = missing data. | | |  |  |  |  |  |  |  |  |

Supplementary table 6. Single-Tissue eQTLs for rs17268364.

| Tissue | Samples | p-value |
| --- | --- | --- |
| Testis | 322 | 8.6×10^-12^ |
| Esophagus - Gastroesophageal Junction | 330 | 1.2×10^-3^ |
| Artery - Aorta | 387 | 1.6×10^-3^ |
| Uterus | 129 | 5.0×10^-3^ |
| Nerve - Tibial | 532 | 1.0×10^-2^ |
| Heart - Left Ventricle | 386 | 1.0×10^-2^ |
| Lung | 515 | 1.0×10^-2^ |
| Prostate | 221 | 3.0×10^-3^ |
| Artery - Tibial | 584 | 3.0×10^-4^ |
| Spleen | 227 | 4.0×10^-5^ |

Data Source: GTEx Analysis Release V8 (dbGaP Accession phs000424.v8.p2)

**Figure legend**

**Supplementary Figure 1. The correlation between mRNA expression of CTLA4 and rs17268364 genotypes**

A. Healthy controls B. systemic lupus erythematosus patients without renal impairment

**Supplementary Figure 2.** The correlation between mRNA expression of *ICOS* and rs17268364 genotypes in SLE patients without renal impairment (A), lupus nephritis patients (B), and SLE patients (C).

**Supplementary Figure 3. Linkage disequilibrium (LD) heatmap of the 24 identified SLE-associated SNPs.**

The Linkage disequilibrium (LD) heatmap of the 24 identified SLE-associated SNPs was generated using genotype data of 103 Chinese Han Beijing individuals from 1000 genome project. The degrees of LD were estimated by CI method using Haploview4.2 (Cambridge, MA, USA) and a standard color scheme (D’/LOD) is used to display the LD pattern.

**Reference**

1. Sun C, Molineros JE, Looger LL, Zhou XJ, Kim K, Okada Y, et al. High-density genotyping of immune-related loci identifies new SLE risk variants in individuals with Asian ancestry. Nat Genet. 2016;48(3):323-30.

2. Ward LD, Kellis M. HaploReg: a resource for exploring chromatin states, conservation, and regulatory motif alterations within sets of genetically linked variants. Nucleic acids research. 2012;40(Database issue):D930-4.
