# Supplementary figures and images for: Lupus susceptibility region containing CTLA4 rs17268364 functionally reduces CTLA4 expression by binding EWSR1 and correlates IFN-α signature
Source: Arthritis Res Ther. 2021 Nov 4;23:279. doi: 10.1186/s13075-021-02664-y (PMC8567630; doi:10.1186/s13075-021-02664-y)

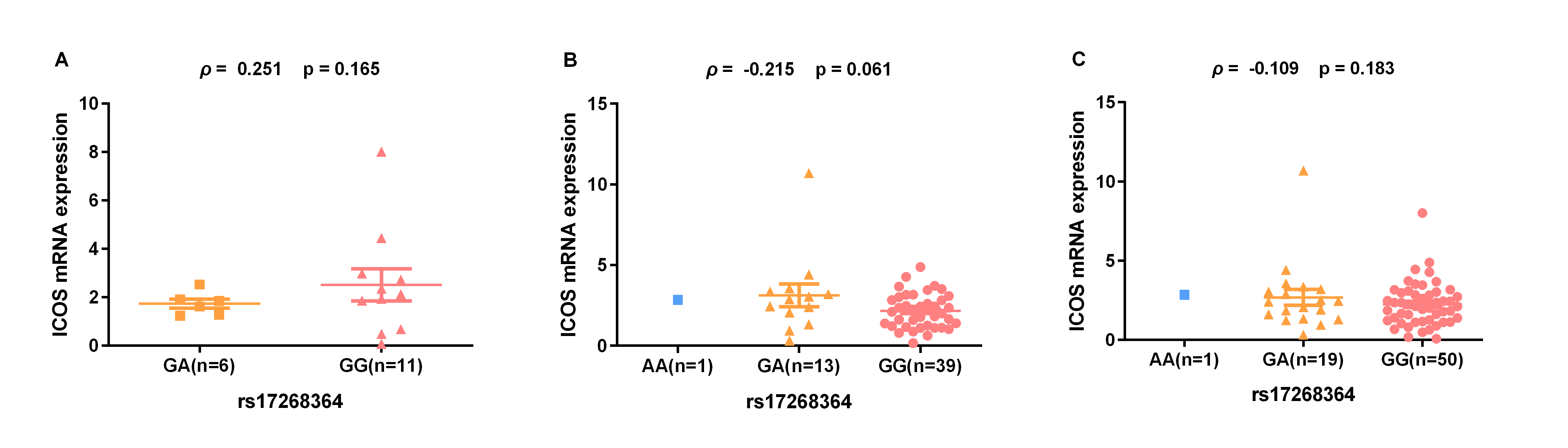

Supplement: Supplementary file 1 — Additional file 1: Supplementary table 1. Demographical information of the cohorts. Supplementary table 2. Association results of SNPs in CTLA4-ICOS region and SLE susceptibility (1). Supplementary Table 3. Synthesized sequences for subcloning into pGL3-promoter. Supplementary Table 4. The sequences of the synthetic double-stranded oligonucleotides for protein mass spectrometry and EMSA. Supplementary table 5. Regulatory chromatin states from DNAse and histone ChIP-Seq (Roadmap Epigenomics Consortium, 2015) (2). Supplementary table 6. Single-Tissue eQTLs for rs17268364. Supplementary Figure 1. The correlation between mRNA expression of CTLA4 and rs17268364 genotypes. A. Healthy controls B. systemic lupus erythematosus patients without renal impairment. Supplementary Figure 2. The correlation between mRNA expression of ICOS and rs17268364 genotypes in SLE patients without renal impairment (A), lupus nephritis patients (B), and SLE patients (C). Supplementary Figure 3. Linkage disequilibrium (LD) heatmap of the 24 identified SLE-associated SNPs. The Linkage disequilibrium (LD) heatmap of the 24 identified SLE-associated SNPs was generated using genotype data of 103 Chinese Han Beijing individuals from 1000 genome project. The degrees of LD were estimated by CI method using Haploview4.2 (Cambridge, MA, USA) and a standard color scheme (D’/LOD) is used to display the LD pattern. [file 13075_2021_2664_MOESM1_ESM.zip › supplementary figure 2.tif]

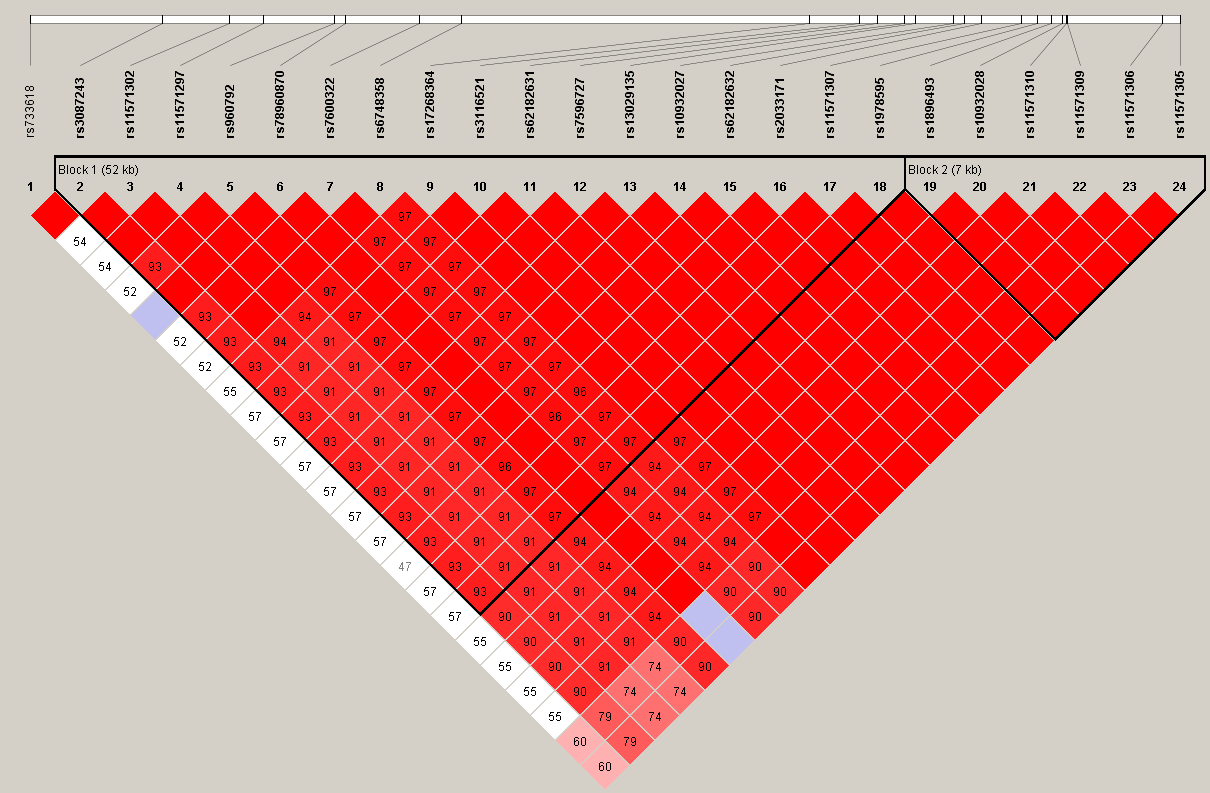

Supplement: Supplementary file 1 — Additional file 1: Supplementary table 1. Demographical information of the cohorts. Supplementary table 2. Association results of SNPs in CTLA4-ICOS region and SLE susceptibility (1). Supplementary Table 3. Synthesized sequences for subcloning into pGL3-promoter. Supplementary Table 4. The sequences of the synthetic double-stranded oligonucleotides for protein mass spectrometry and EMSA. Supplementary table 5. Regulatory chromatin states from DNAse and histone ChIP-Seq (Roadmap Epigenomics Consortium, 2015) (2). Supplementary table 6. Single-Tissue eQTLs for rs17268364. Supplementary Figure 1. The correlation between mRNA expression of CTLA4 and rs17268364 genotypes. A. Healthy controls B. systemic lupus erythematosus patients without renal impairment. Supplementary Figure 2. The correlation between mRNA expression of ICOS and rs17268364 genotypes in SLE patients without renal impairment (A), lupus nephritis patients (B), and SLE patients (C). Supplementary Figure 3. Linkage disequilibrium (LD) heatmap of the 24 identified SLE-associated SNPs. The Linkage disequilibrium (LD) heatmap of the 24 identified SLE-associated SNPs was generated using genotype data of 103 Chinese Han Beijing individuals from 1000 genome project. The degrees of LD were estimated by CI method using Haploview4.2 (Cambridge, MA, USA) and a standard color scheme (D’/LOD) is used to display the LD pattern. [file 13075_2021_2664_MOESM1_ESM.zip › supplementary figure 3.png]
